# Neuroprotective potential of quercetin in Alzheimer’s disease: targeting oxidative stress, mitochondrial dysfunction, and amyloid-β aggregation

**DOI:** 10.3389/fphar.2025.1593264

**Published:** 2025-06-11

**Authors:** Mohd Adnan, Arif Jamal Siddiqui, Fevzi Bardakci, Malvi Surti, Riadh Badraoui, Mitesh Patel

**Affiliations:** ^1^ Department of Biology, College of Science, University of Ha’il, Ha’il, Saudi Arabia; ^2^ King Salman Center for Disability Research, Riyadh, Saudi Arabia; ^3^ Research and Development Cell (RDC), Parul University, Vadodara, Gujarat, India; ^4^ Department of Biotechnology, Parul Institute of Applied Sciences, Parul University, Vadodara, Gujarat, India

**Keywords:** quercetin, Alzheimer’s disease, amyloid-beta, neuroprotection, oxidative stress, Aβ aggregation, SH-SY5Y cells

## Abstract

**Introduction:**

Alzheimer’s disease (AD) is a progressive neurodegenerative disorder characterized by amyloid-beta (Aβ) peptide accumulation, oxidative stress, mitochondrial dysfunction and cholinergic deficits, all of which contribute to neuronal damage and cognitive decline.

**Methods:**

This study investigated the neuroprotective potential of quercetin, a natural flavonoid, in human neuroblastoma SH-SY5Y cells exposed to Aβ-induced toxicity. Various assays were conducted to evaluate cell viability, reactive oxygen species (ROS) levels, mitochondrial membrane potential (ΔΨm), acetylcholinesterase (AChE) activity and Aβ aggregation.

**Results:**

Quercetin significantly enhanced cell viability and reduced oxidative stress by lowering intracellular ROS levels. It preserved mitochondrial integrity by stabilizing ΔΨm and inhibited AChE activity, thereby supporting cholinergic function. Additionally, quercetin reduced Aβ aggregation and the formation of toxic amyloid fibrils.

**Discussion:**

These findings suggest that quercetin confers neuroprotection by targeting multiple pathological mechanisms involved in AD, including oxidative stress, mitochondrial dysfunction, AChE activity and Aβ aggregation. Quercetin demonstrates promise as a natural therapeutic agent for the treatment of AD. However, further *in-vivo* investigations and clinical studies are warranted to validate these findings and explore its translational potential.

## Introduction

Alzheimer’s disease (AD) is a major challenge of modern medicine that is characterized by progressive neurodegeneration and cognitive impairment. AD is a progressive neurological condition that erodes cognitive functions and memory. Despite progress, the exact pathogenesis of AD is still not well understood and this has led to challenges in developing effective therapies (Choe et al., 2022; Leventhal et al., 2024). The development of AD is often linked to the increasing deposition of amyloid-beta (Aβ) plaques and neurotoxic tangles of tau protein, which impair neural function and eventually result in neuronal cell death (Janelidze et al., 2020; Griciuc and Tanzi, 2021). Now, AD has come to be understood as a multi-faceted disease reliant on an interplay between genetic, environmental and lifestyle factors. For example, all the genetic risk factors identified to date in AD act by promoting synaptic degeneration or increasing the levels of toxic Aβ oligomers, with the apolipoprotein E4 (APOE4) allele best characterized (Jackson et al., 2019). In addition, cerebrovascular health has been shown to have a complex association with cognitive decline, since vascular risk factors including hypertension and diabetes have also been implicated in the early progression of AD (Pluta et al., 2021; Ferrari-Souza et al., 2024). Likewise, the relationship between neuroinflammation and neurodegeneration is essential to consider, since activated microglia and astrocytes create the inflammatory response that causes damage to neurons (Johnson et al., 2020; Griciuc and Tanzi, 2021).

It has been recently suggested the possibility that oxidative stress and metabolic dysregulation lead to AD. High redox status caused by sustained ROS can result in tau hyperphosphorylation and neurofibrillary tangle formation, important elements of AD pathology (Sundstrom et al., 2018). Disruption of energy metabolism on a more granular scale, particularly in the context of microglia and astrocytes, has also been linked to cognitive impairment, thus suggesting that this metabolic shift may underlie and perhaps precede the neurodegenerative process itself (Johnson et al., 2020; Lewczuk et al., 2020). Furthermore, the link between sleep disturbances and AD has gained traction, with emerging evidence indicating that cholinergic dysfunction may induce sleep disturbances, thereby perpetuating cognitive decline (Choe et al., 2022). Such complexity of AD requires integrative approaches to better determine its etiology and develop effective therapies. As further complexity in the AD model is being researched, it is evident that a more complete understanding of AD pathology is needed to advance the development of disease modification strategies and outcomes in AD patients.

Quercetin is a flavonoid that is ubiquitous in fruits and vegetables and has been studied for its potential neuroprotective properties. Recent findings suggested that quercetin might inhibit Aβ production through the modulation of β-secretase activity resulting in decreased level of this neurotoxic peptide (Mugundhan et al., 2024). Moreover, quercetin beyond its role as a neuroprotective agent due to its antioxidant properties, it may antagonizes the oxidative stress caused by Aβ toxicity (Croce et al., 2012; Mattioli et al., 2019). Quercetin exhibits neuroprotective effects through multiple molecular pathways. It enhances brain-derived neurotrophic factor (BDNF) expression, supporting neuronal survival and synaptic plasticity, thus aiding cognitive function (Rahvar et al., 2018). It also regulates mitochondrial integrity and apoptosis by modulating Bcl-2, Bax, cytochrome c, and caspase-3, helping prevent neurodegeneration (Zhang et al., 2024). Quercetin activates the PI3K/Akt signaling pathway, promoting anti-apoptotic activity and neuronal recovery after injury (Chang et al., 2014; Jeon et al., 2017). Moreover, it inhibits neuroinflammation by downregulating NF-κB, reducing pro-inflammatory cytokine production (Granado-Serrano et al., 2012). Through its antioxidant properties, quercetin enhances cellular defense mechanisms and resilience against oxidative stress, contributing to overall neuronal health (Costa et al., 2013; Bayazid and Lim, 2022). Due to its interaction with a number of signaling pathways, including pathways that are implicated in apoptosis and inflammation, quercetin is a promising candidate for therapy in AD (Petry et al., 2020).

Thus, the aim of this study was to assess the neuroprotective effect of quercetin against Aβ peptide-induced toxicity in human neuroblastoma SH-SY5Y cells. In the present study, we utilized this well-characterized cellular model to investigate the neuroprotective potential of quercetin by evaluating its effects on oxidative stress, mitochondrial membrane stability, AChE inhibition and Aβ aggregation, which are key contributors to Aβ-induced neurotoxicity. The neuroprotective properties of several compounds, such as polyphenols and peptides, have been demonstrated against Aβ toxicity by different mechanisms, mainly inhibition of Aβ aggregation or modulation of apoptotic pathways (González-Sanmiguel et al., 2020; Mallesh et al., 2024). This study aims to clarify the neuroprotective potential of the natural flavonoid quercetin and, as such, to add to advances in therapeutic strategies for AD and other neurodegenerative disorders.

## Materials and methods

### Chemicals

Dulbecco’s Modified Eagle Medium (DMEM) and fetal bovine serum (FBS) were purchased from Gibco, United States. Key experimental reagents, including 5,5′-dithiobis-(2-nitrobenzoic acid) (DTNB), 2′,7′-dichlorofluorescein diacetate (DCFDA), Aβ 1-42 (Aβ_1-42)_ peptide and MTT were obtained from Merck-Sigma Aldrich. All remaining chemicals were acquired from HiMedia^®^, India.

### Antioxidant assay

#### DPPH assay

The DPPH assay based on (Brand-Williams, 1999) was used to determine quercetin’s radical scavenging capacity. A freshly prepared DPPH solution (0.78 mg/20 mL) was reacted with quercetin (50–300 μM). Briefly, 1 mL 0.1 mM DPPH was added to 100 μL quercetin, incubated for 15 min in the dark, and absorbance measured at 517 nm. As a positive control, ascorbic acid was used. The calculation of % inhibition was carried out via following equation.

$$\text{Inhibition of DPPH radical }\left(\%\right)=\text{Ab }–\text{ As}/\text{Ab}\times 100$$

Where, Ab = absorbance of the blank (DPPH solution without sample) and As = absorbance of the sample (DPPH solution with quercetin).

### FRAP (ferric-reducing antioxidant power) assay

The Ferric Reducing Antioxidant Power (FRAP) method, as detailed by (Benzie and Strain, 1996) was further utilized to quantify the antioxidant potential of quercetin. The initial mixture of assay was performed with the combination of 30 mM FeCl_3_·6H_2_O, 10 mM 2,4,6-tripyridyl-S-triazine and 150 mM acetate buffer (pH-3.6) in 40 mM hydrochloric acid at the ratio of 10:1:1 at the room temperature. Then, 3.95 mL of freshly prepared FRAP reagent, and 5 μL of differing concentrations of quercetin (50–300 μM) was added to this mixture. The mixture was left to incubate at room temperature for 30 min. With quercetin in a solution, ferric ions are reduced to ferrous ions, where the ferrous ions are chelated with TPTZ to produce the blue-colored ferrous-TPTZ complex. Subsequently, the absorption was taken at 593 nm. As a positive control, ascorbic acid was used.

### AChE inhibition

The Ellman spectrophotometric method was employed to quantify cholinesterase inhibitory activity (Ellman et al., 1961). In this assay, 3 mL 0.1 M Tris-HCl buffer (pH - 8.0) was mixed with 20 μL AChE solution (3 U/mL). Then, 100 μL solution of different concentrations of quercetin (50–300 μM) were included in the solution, incubated at room temperature for 15 min. After this incubation, 50 μL of teramethylbenzothiazole-2-thione (DTNB) was added (3 mM). Initiation of the reaction was occurred by adding 50 μL of 15 mM acetylthiocholine iodide (AChI), when a yellow colour was observed. Galantamine was served as the positive control. Measurements of absorbance were taken at 412 nm with a UV-visible spectrophotometer (UV-2600, Shimadzu, Japan). Enzyme inhibition percentages were calculated via comparing the enzyme activities with respect to negative control as per following equation:

$$\text{Inhibition }\left(\%\right)=A0\;–\;A1\times 100$$

Where, A0 = Absorbance of the control (reaction mixture without quercetin). A1 = Absorbance of the test sample (reaction mixture with quercetin).

### Thioflavin T (ThT) assay

This method is often utilized as an assay to determine the kinetics of fibrillogenic in the context of amyloid formation. Thioflavin T (ThT) binds to amyloid fibrils and fluoresces, and thus facilitates monitoring of *in-vitro* amyloid fibril aggregation (Xue et al., 2017). Different quercetin concentrations were combined with 20 μM of Aβ_1-42_ in a volume of 40 μL, and incubated at 37°C for 24 h. Then, 100 μL of ThT solution was added to the solution after incubation. After a 30 min incubation, the fluorescence intensity of the samples were measured using a FP-6200 spectrofluorometer (Shimadzu, RF-5000). The detection wavelengths were 450 nm (excitation) and 483 nm (emission). Galantamine with Aβ_1-42_ was served as the positive control for comparison.

### Neuroprotective effects of quercetin

#### Preparation of quercetin stock solution

The test samples were prepared as stock solutions in culture media at a concentration of 500 µM and sterilized through a 0.2-micron PES membrane syringe filter. In distinct wells, different volumes of the filtered stock solution were added to achieve final concentrations ranging from 50 to 300 μM. As quercetin is sparingly soluble in water, the appropriate solubilization was carried out by initially dissolving it in dimethyl sulfoxide (DMSO) to prepare a concentrated stock solution. This stock was then diluted with the respective culture medium to achieve the desired working concentrations for cell assays. The final concentration of DMSO in all treatments, including control groups, were kept below 0.1% to avoid any solvent-induced cytotoxic effects.

### Aβ_1-42_ stock solution and working solution

To prepare the Aβ 1-42 (Aβ_1-42_) stock solution, it was first dissolved in distilled water to achieve a 1 mM concentration. Subsequently, a working solution of 100 μM was made by diluting the stock in cell culture medium supplemented with 10% FBS. This working solution was then applied to the wells, resulting in final Aβ_1-42_ concentrations spanning 0.625–20 µM.

### Cell culture

The SH-SY5Y cell line was sourced from the National Centre for Cell Science (NCCS) in Pune, India. The studies were performed with SH-SY5Y neuroblastoma cells cultured in DMEM containing non-essential amino acids (1X) and 10% FBS under 5% CO_2_ at 37°C and were used for experiments when 80% of confluence was reached.

### Assessment of cell viability

A modified MTT assay was used to evaluate cell viability (Kenchappa et al., 2023). Centrifugation at 300 × g was performed to collect the cells and resuspended in DMEM-HG medium. The suspension was seeded at a concentration of about 15,000 cells in 200 μL. Then, 200 μL of a suspension of cells were added to each well in a 96-well microtiter plate and then incubated under 5% CO_2_ in a humidified environment for 24 h at 37°C. The medium was then carefully replaced with 200 μL of various concentrations of quercetin. The plate was placed back in the incubator for an additional 24 h under identical conditions. Following this incubation, the spent medium was carefully aspirated and 200 μL of fresh medium containing 10% MTT reagent (final concentration - 0.5 mg/mL) was introduced into each well. The plate was then incubated at 37°C with 5% CO_2_ for 3 h to allow for formazan crystal formation. After incubation, the medium was gently removed while ensuring the crystals remained undisturbed. To dissolve the formazan, 100 μL of DMSO was added to each well and the plate was subjected to gentle agitation using a gyratory shaker. The optical density at 570 nm and 630 nm was measured using a microplate reader to evaluate cell viability. The absorbance of the background was subtracted, and percentage of growth inhibition was determined. The IC_50_ value was calculated from the dose-response curve based on the concentration of test compound required to inhibit it by 50%.

### Assessment of cytoprotective activity

The protective role of quercetin against Aβ_1-42_ induced toxicity was assessed using MTT assay, as described before (Dalberto et al., 2020). As per our prior methodology, cells were placed into 96-well plates and left to incubate for 24 h. After incubation, the spent medium was aspirated and 100 μL of different concentrations of quercetin was added to the wells and initiated further incubation at 37°C with 5% CO_2_ for 4 h. Then after treating with quercetin, 100 μL of β-Amyloid (10 μM) or (20 μM) were added to the corresponding wells, and again plate was incubated for 48 h at 37°C with 5% CO_2_. After the initial incubation, the spent culture medium was carefully removed from each well. A fresh 200 μL aliquot of medium containing 10% MTT reagent was then added to the wells. The plate was incubated for 3 h at 37°C under a 5% CO_2_ atmosphere to allow formazan formation. Following incubation, the medium was gently aspirated, ensuring the retention of MTT-derived crystals. To fully solubilize the formazan, 100 μL of DMSO was added to each well, and the plate was gently agitated using a gyratory shaker. Optical density readings were recorded at 570 nm and 630 nm using a microplate reader.

### Mitochondrial membrane potential (Δψm) assay

Cells at a density of 3 × 10^5^ cells in 2 mL were plated in 6-well plates, and incubated overnight at 37°C in a CO_2_-controlled environment to facilitate attachment. The following day, the culture medium was replaced with fresh medium supplemented with 100 µM quercetin, and cells were maintained under the same conditions for 4 h. After 24 h, cells were subjected to 20 μM Aβ_1-42_ treatment, and incubated for an additional 96 h. At the end of the treatment, cells were collected and centrifuged at 300 × g for 5 min at room temperature. The resulting pellet was washed twice with PBS to remove residual medium. Next, the cells were resuspended in 0.5 mL of freshly prepared JC-1 staining solution, and gently mixed to ensure an even distribution. The suspension was then incubated at 37°C in a CO_2_ incubator for 10–15 min. Following staining, cells were washed using a 1X assay buffer, resuspended, and immediately subjected to flow cytometry analysis. Furthermore, mitochondrial membrane potential (Δψm) in SH-SY5Y cells were assessed using JC-1 staining, via fluorescence imaging to detect changes in red (aggregates) and green (monomers) fluorescence, serving as an indicator of mitochondrial health, and allowing differentiation between live (polarized), and dead or apoptotic (depolarized) cells (Chang et al., 2013; Kim and Xue, 2020).

### ROS estimation by H_2_DCFDA staining

Intracellular ROS levels were assessed by culturing cells in 6-well plates at a density of 3 × 10^5^ cells per well in 2 mL of medium, followed by overnight incubation at 37°C in a CO_2_-enriched environment. The next day, cells underwent a 4 h pre-treatment with 100 µM quercetin. This was followed by exposure to 20 μM Aβ_1-42_ for a duration of 96 h. After the treatment period, 5 μL of a 10 μM H_2_DCFDA solution was introduced into each well and the cells were incubated at 37°C for 1 h. Subsequently, cells were detached using trypsin, transferred into 5 mL tubes, and processed for flow cytometric analysis. Following centrifugation at 300 × g for 5 min at room temperature, the cell pellets were washed twice with PBS, and resuspended in 500 μL of pre-warmed Dulbecco’s Phosphate-Buffered Saline (DPBS). Flow cytometry was conducted using a 488 nm excitation wavelength, with fluorescence emission detected at 525 nm (FL1) (Chang et al., 2013; Kim and Xue, 2020).

### Statistical analysis

All results are expressed as the mean ± standard deviation (SD) from at least three independent experiments. The statistical significance of the antioxidant potential of quercetin, as determined by DPPH and FRAP assays, were analyzed using two-way ANOVA followed by Bonferroni’s multiple comparison test. For all other experimental assays, one-way ANOVA followed by Dunnett’s multiple comparison test was employed to assess statistical significance. A p-value of less than 0.05 was considered statistically significant. All analyses were performed using GraphPad Prism software version 8.0 (GraphPad Software, Inc., United States).

## Results

### Antioxidant activity

**FIGURE 1:**
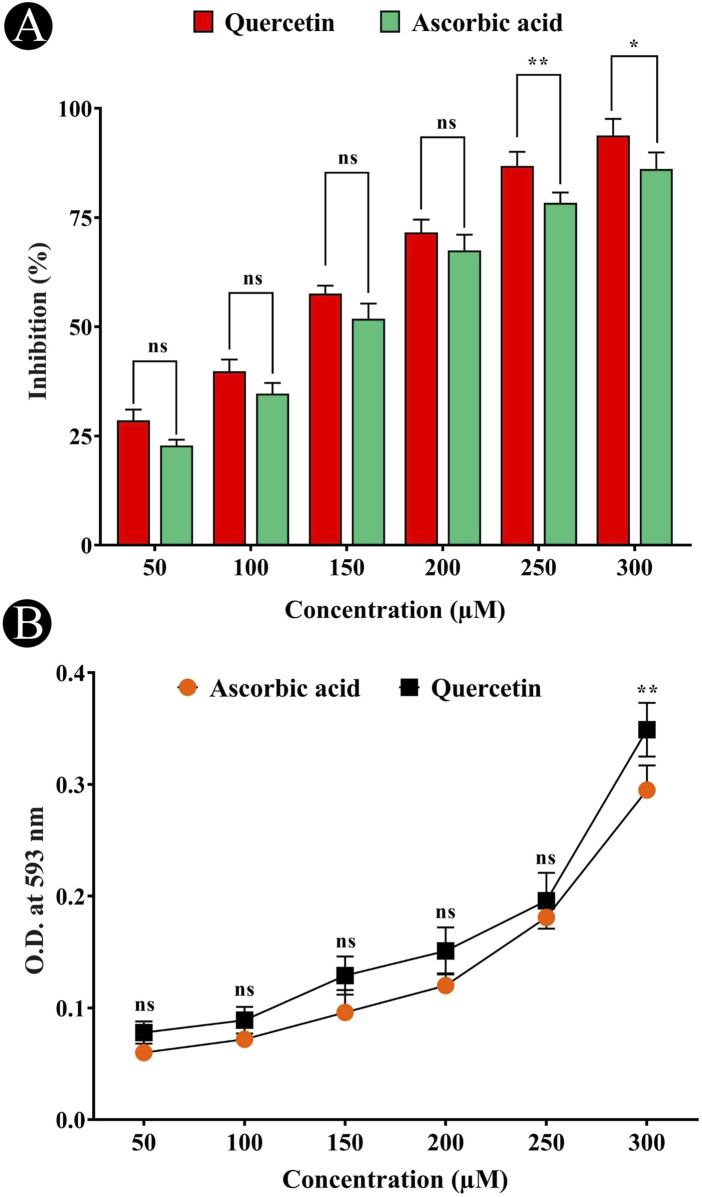
Antioxidant potential of quercetin evaluated using *in-vitro* assays. **(A)** DPPH (2,2-diphenyl-1-picrylhydrazyl) radical scavenging assay showing dose-dependent antioxidant activity of quercetin. **(B)** Ferric reducing antioxidant power (FRAP) assay demonstrating the electron-donating capacity of quercetin across tested concentrations. Data are presented as mean ± standard deviation (SD) from three independent experiments. Statistical significance is denoted as follows: ns (not significant) > 0.05, *p < 0.05, **p < 0.005, ***p < 0.0005.

Oxidative stress markers are playing a role in the development of neurodegeneration, causing to cellular damage and death. The DPPH and FRAP assays were used to evaluate antioxidant activity. DPPH assay measured absorbance at 515 nm to detect free radicals, and similar measurements were made for FRAP assay at 593 nm. A shift from deep violet to pale yellow observed in the DPPH assay are indicative of antioxidant activity, while the FRAP assay demonstrated an increase in the Fe^2+^-TPTZ complex, suggesting effective donation of electrons by the antioxidants. The quercetin showed concentration dependent antioxidant activity. These observations suggest that quercetin is an affective scavenger of free radicals and electron donor, leading to their strong antioxidant qualities (Figures 1A, B).

### Inhibition of AChE and the potential for Thioflavin T (ThT) binding

**FIGURE 2:**
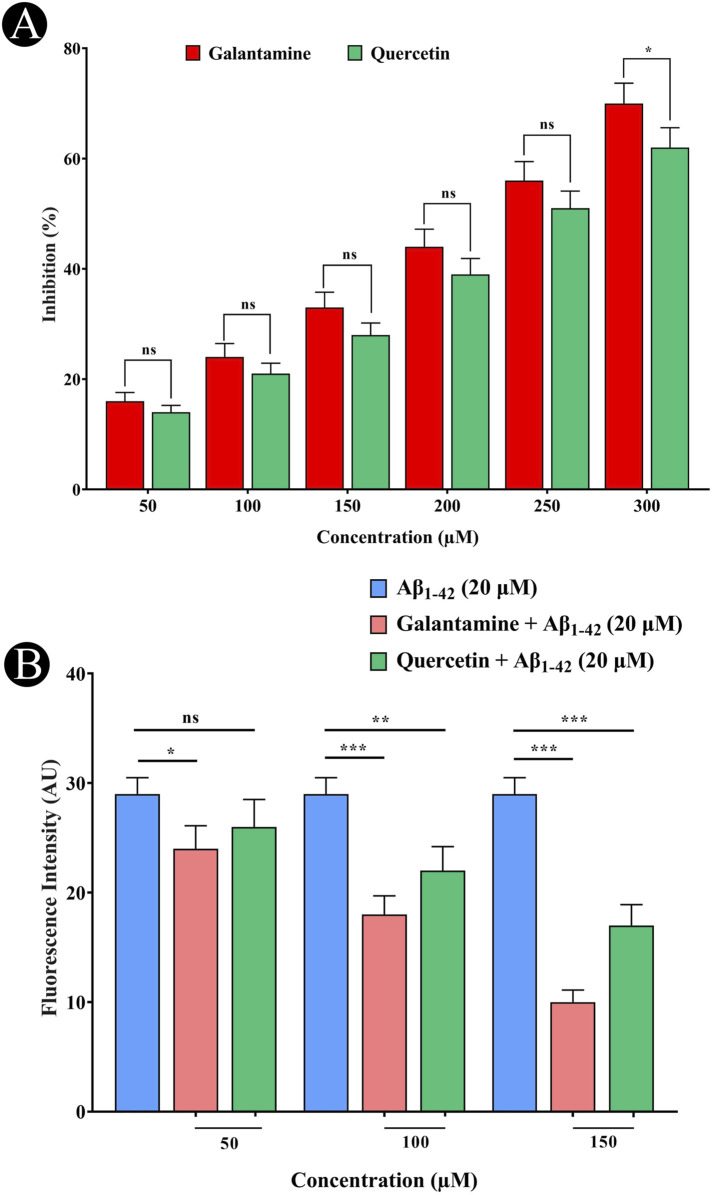
Evaluation of AChE inhibitory activity and inhibition of amyloid aggregation by quercetin. **(A)** Inhibitory effect of quercetin on AChE activity, compared with the standard compound galantamine. **(B)** Thioflavin-T fluorescence assay showing inhibition of Aβ_1-42_ aggregation in the presence of increasing concentrations of quercetin and galantamine. Data are presented as mean ± standard deviation (SD) from three independent experiments. Statistical significance is denoted as follows: ns (not significant) > 0.05, *p < 0.05, **p < 0.005, ***p < 0.0005.

The AChE inhibitory activity of quercetin is shown in Figure 2A, which demonstrated the concentration-dependent inhibition of AChE by quercetin. Phenolic and flavonoid compounds found in nature have been identified to have neuroprotective actions by inhibiting AChE activity, which represents an effective strategy for treating AD. Further evaluation of the neuroprotective potential of quercetin was performed using the ThT fluorescence assay (Figure 2B). The results showed that quercetin inhibited the binding of ThT to amyloid, suggesting that it could inhibit amyloid-β aggregation effectively.

### MTT assay and cell viability

**FIGURE 3:**
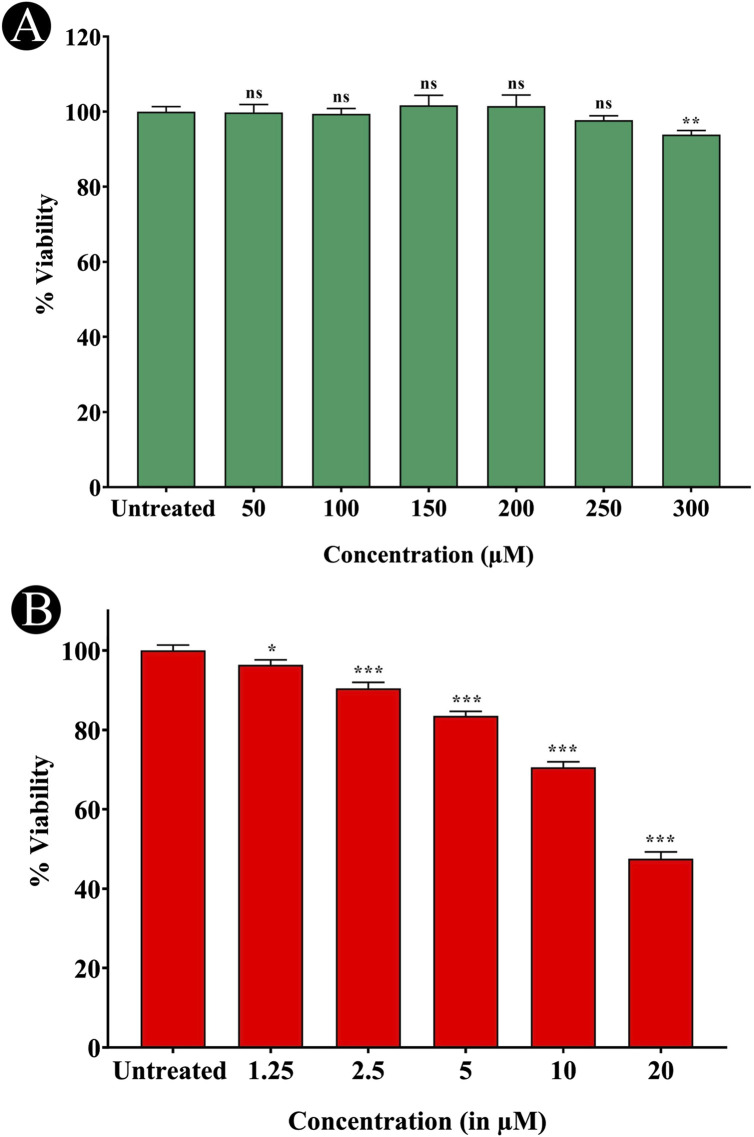
Assessment of cytotoxic effects of quercetin and Aβ_1-42_ on SH-SY5Y Cells. **(A)** MTT assay showing cell viability following treatment with various concentrations of quercetin, indicating its non-cytotoxic nature. **(B)** MTT assay demonstrating cytotoxic effects of Aβ_1-42_ on SH-SY5Y cells. Data are presented as mean ± standard deviation (SD) from three independent experiments. Statistical significance is denoted as follows: ns (not significant) > 0.05, *p < 0.05, **p < 0.005, ***p < 0.0005.

**FIGURE 4:**
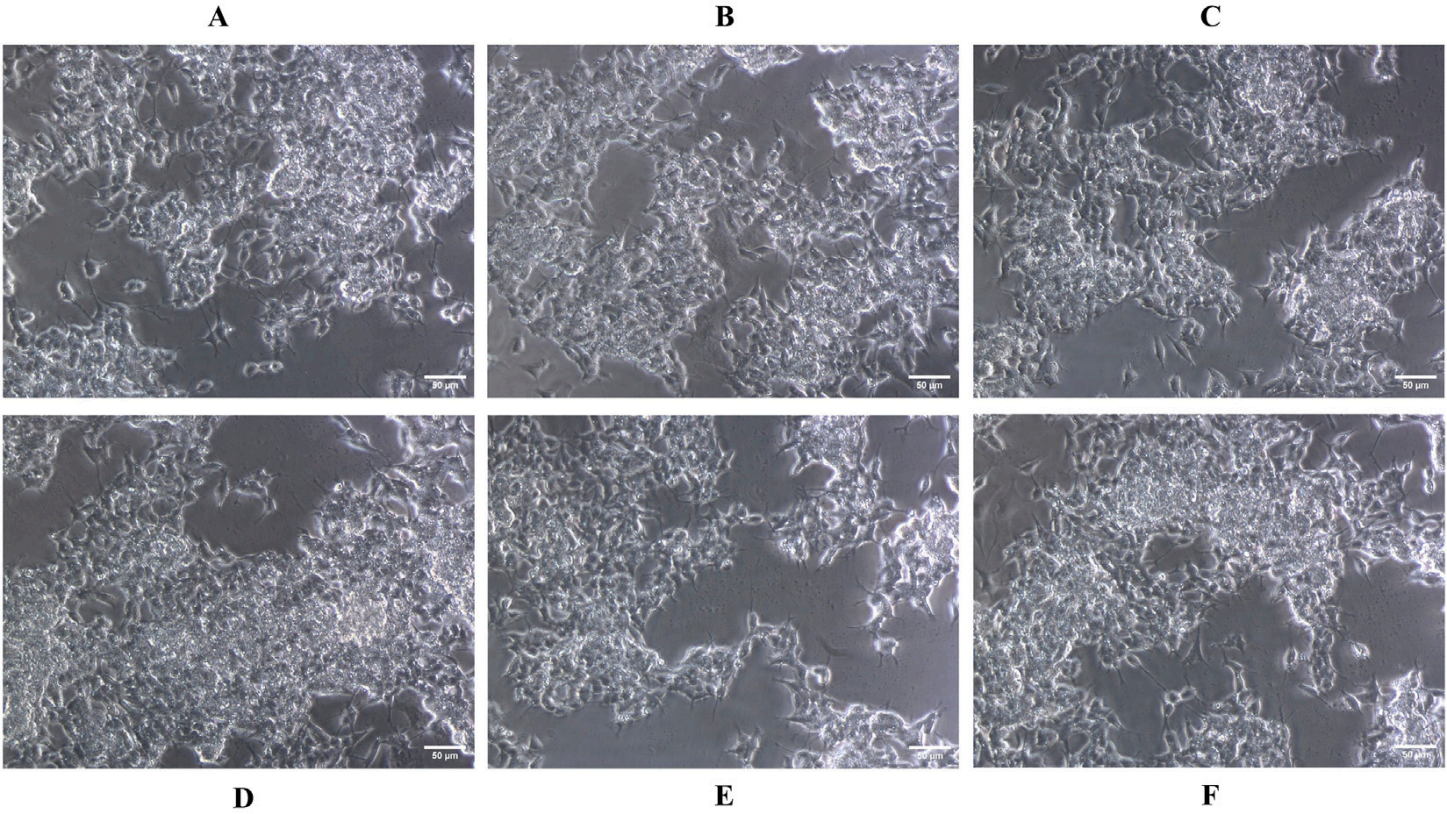
Microscopic images showing the morphological assessment of SH-SY5Y cells treated with quercetin at various concentrations. **(A)** Untreated control, **(B)** 100 μM, **(C)** 150 μM, **(D)** 200 μM, **(E)** 250 μM, and **(F)** 300 µM. No significant morphological alterations were observed, indicating the non-toxic nature of quercetin up to 300 µM.

**FIGURE 5:**
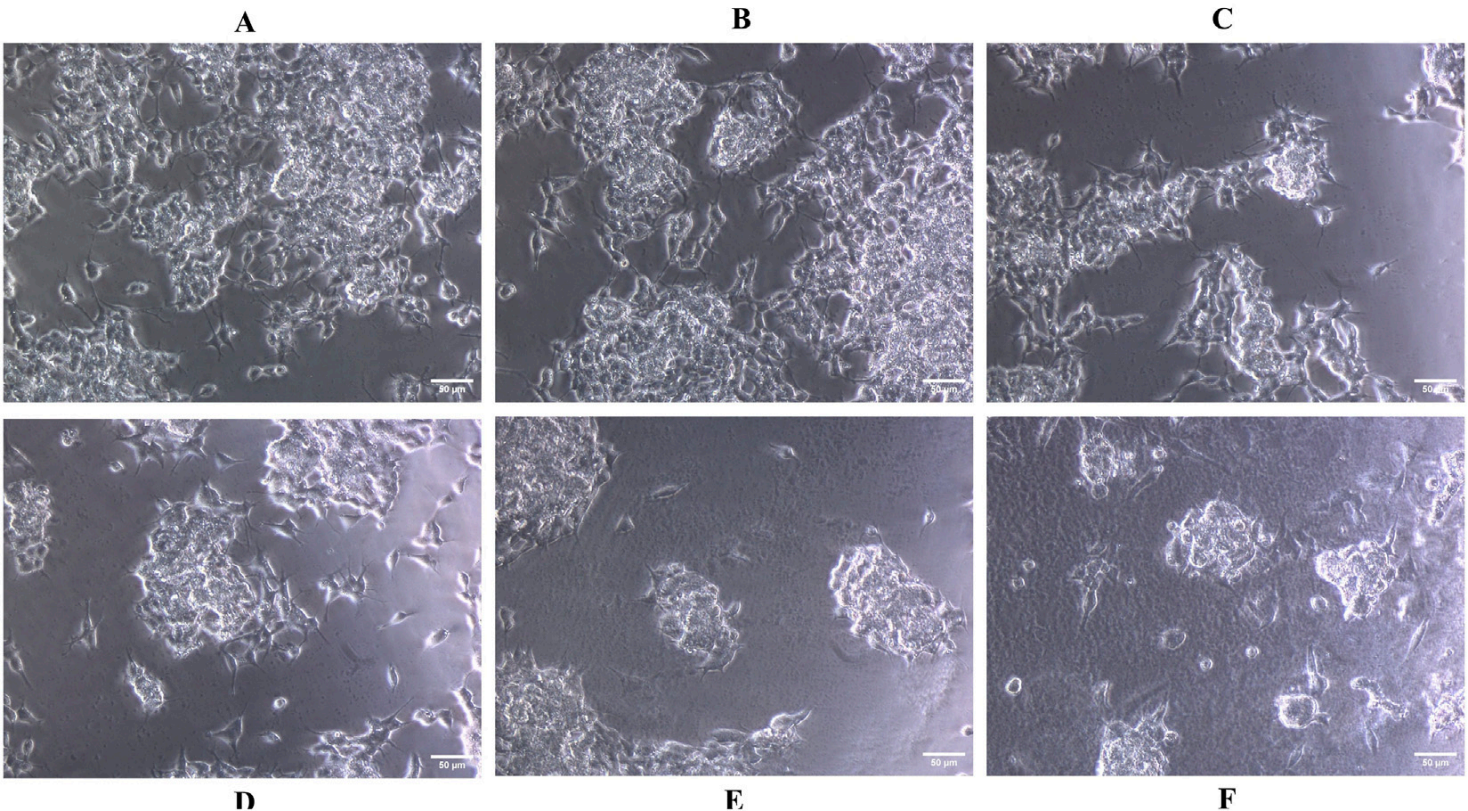
Microscopic images showing dose-dependent morphological changes in SH-SY5Y cells following Aβ_1-42_ treatment. **(A)** Untreated control, **(B)** 1.25 µM, **(C)** 2.5 µM, **(D)** 5.0 µM, **(E)** 10 μM, and **(F)** 20 µM. Increasing Aβ_1-42_ concentrations resulted in pronounced cell shrinkage, rounding and detachment, indicative of neurotoxic effects.

**FIGURE 6:**
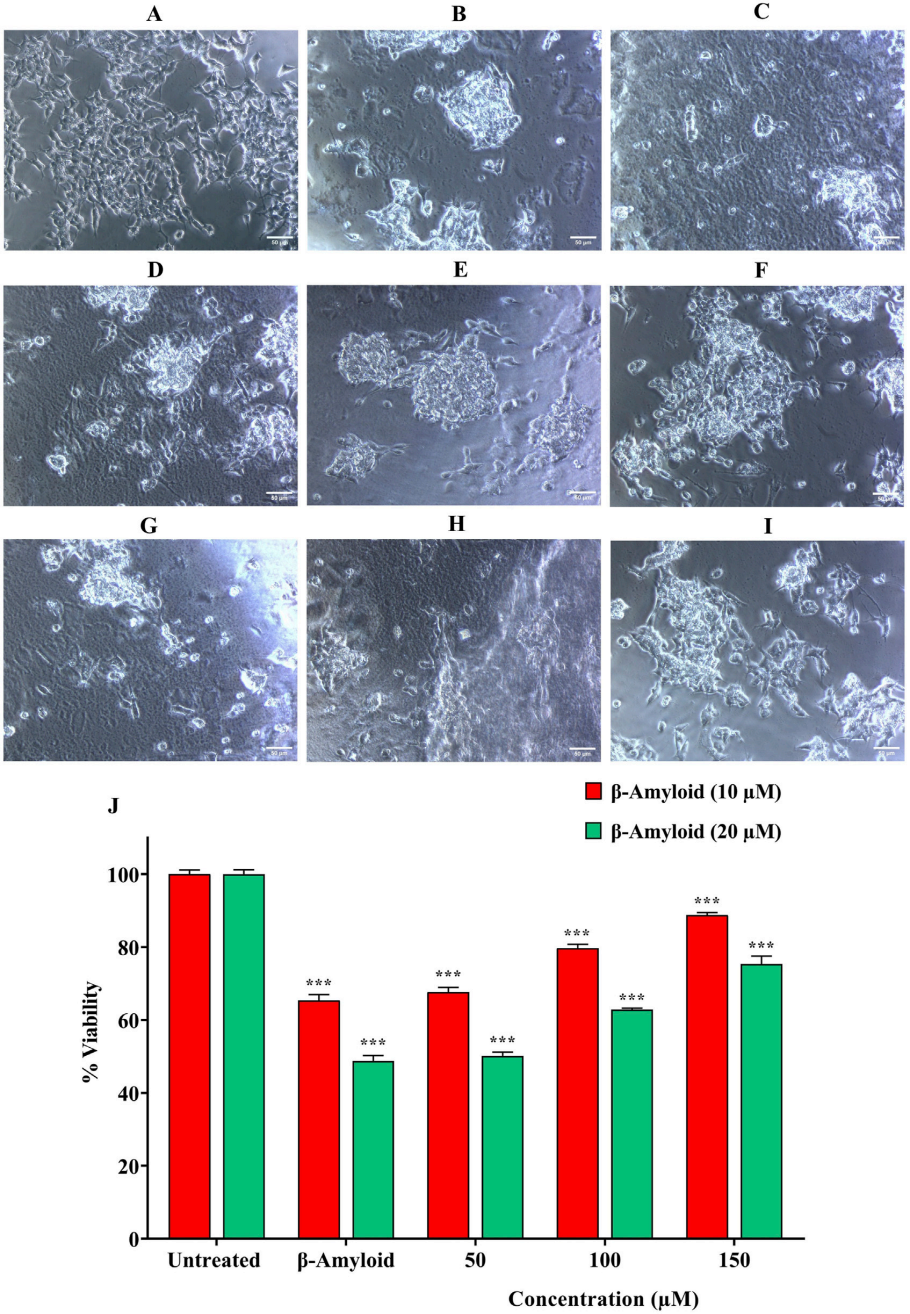
Combined microscopic and MTT assay-based evaluation of SH-SY5Y cells to assess the neuroprotective effects of quercetin against Aβ_1-42_-induced cytotoxicity. Treatment groups include: **(A)** Untreated control, **(B)** Aβ_1-42_ (10 µM), **(C)** Aβ_1-42_ (20 µM), **(D–F)** Aβ_1-42_ (10 µM) co-treated with quercetin (50, 100, 150 µM), and **(G–I)** Aβ_1-42_ (20 µM) co-treated with quercetin (50, 100, 150 µM). **(J)** Quantitative evaluation of neuroprotective effect of quercetin against Aβ_1-42_-induced cytotoxicity using the MTT assay. Data are presented as mean ± standard deviation (SD) from three independent experiments. Statistical significance is denoted as follows: ns (not significant) > 0.05, *p < 0.05, **p < 0.005, ***p < 0.0005. Quercetin co-treatment visibly preserved cell morphology and viability compared to Aβ_1-42_-only groups.

The impact of quercetin on SH-SY5Y cell viability was assessed across a range of concentrations (50–300 μM). No substantial alterations in either cell viability was observed, even at high concentrations reaching 300 μM (Figure 3A). The cytotoxicity of quercetin against SH-SY5Y cells were further evaluated through microscopic observation. The results indicated that quercetin treatment did not induce any noticeable changes in cell shape, size or morphology, suggesting no significant cytotoxic effects under the tested conditions. The cells maintained their typical neuronal morphology with intact cell membranes, and normal adherence properties, further supporting the biocompatibility of quercetin at the examined concentrations. These results suggest a low level of quercetin-induced cytotoxicity in SH-SY5Y cells (Figures 4A–F). In contrast, Aβ_1-42_ exhibited dose-dependent cytotoxicity in SH-SY5Y cells, when it was checked from 1.25 to 20 μM concentrations. The IC_50_ value of Aβ_1_–_42_ in SH-SY5Y cells were determined to be 18.54 μM. Notably, 20 μM Aβ_1-42_ induced significant cytotoxicity, characterized by cell loss, shrinkage and altered morphology, as visualized microscopically (Figures 3B, 5A–F). To explore whether quercetin possesses neuroprotective abilities, SH-SY5Y cells were exposed to Aβ_1-42_ (10 and 20 μM) in combination with varying concentrations of quercetin (50, 100 and 150 μM) for 24 h. The MTT assay was subsequently used to evaluate cell viability. Consistent with previous observations, Aβ_1-42_ treatment alone resulted in a dose-related decrease in cell viability. However, co-treatment with quercetin significantly improved cell survival compared to cells exposed to Aβ_1-42_ alone (10 and 20 μM), demonstrating a clear neuroprotective effect. In the presence of 10 μM Aβ_1-42_, cell viability decreased to 65.30%. Upon treatment with quercetin, cell viability improved to 67.64% at 50 μM (a 3.57% increase), 79.69% at 100 μM (a 22.06% increase), and 88.73% at 150 μM (a 35.84% increase) compared to Aβ_1-42_-treated cells. Similarly, in the presence of 20 μM Aβ_1-42_, cell viability was reduced to 48.89%, which increased to 50.17% at 50 μM (a 2.61% increase), 62.84% at 100 μM (a 28.56% increase) and 75.37% at 150 μM quercetin treatment (a 54.14% increase), respectively. These results indicate a dose-dependent neuroprotective effect of quercetin against Aβ-induced cytotoxicity (Figures 6A–J).

### Effect of quercetin on ROS production

**FIGURE 7:**
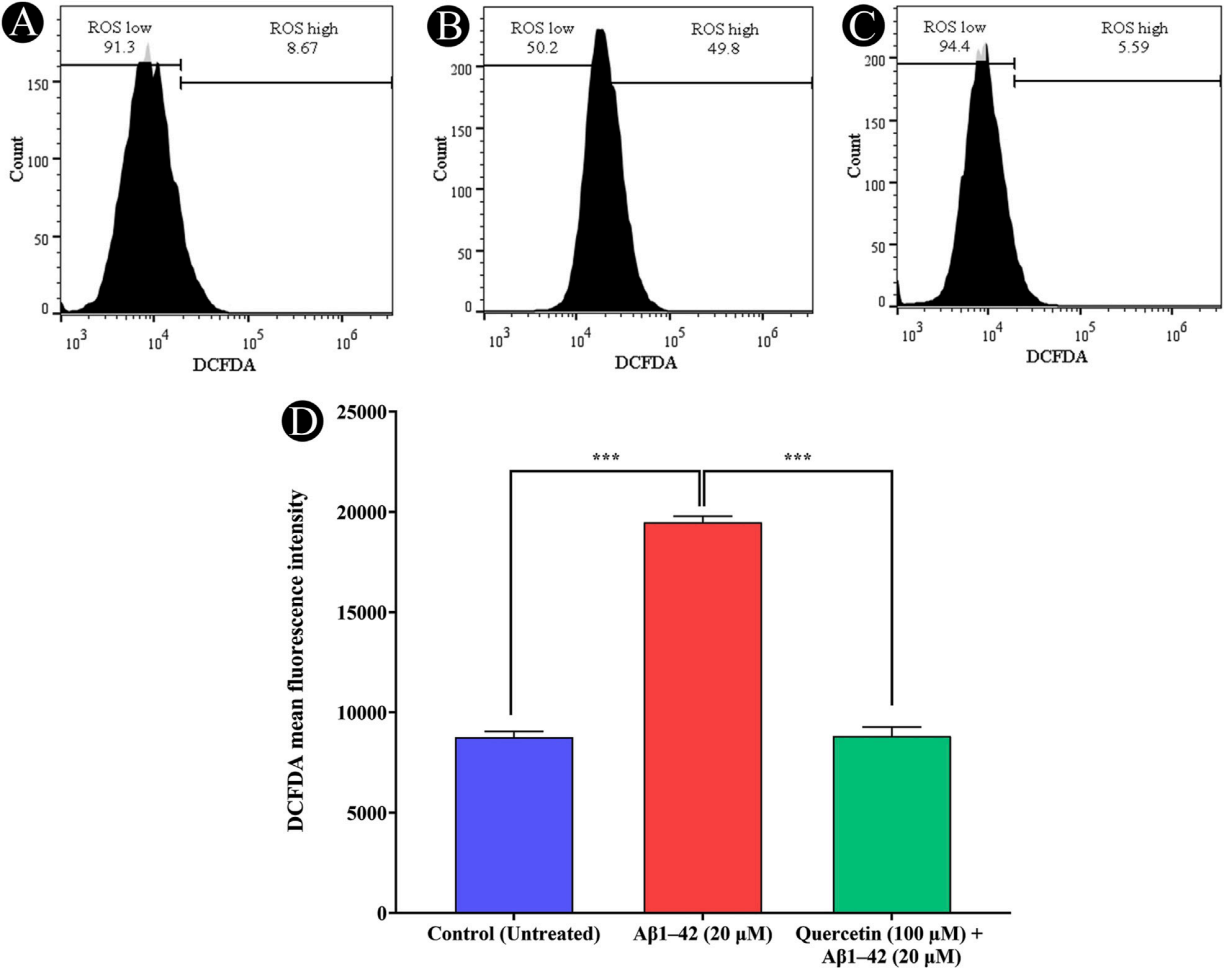
Detection of intracellular ROS generation using H2DCFDA staining and flow cytometry. **(A)** Untreated control cells exhibiting baseline ROS levels, **(B)** Cells treated with Aβ_1-42_ (20 µM), showing increased ROS production, **(C)** Cells pre-treated with quercetin (100 µM) prior to Aβ_1-42_ (20 µM) exposure, demonstrating a reduction in ROS levels, **(D)** Quantitative assessment of H_2_DCFDA fluorescence intensity across treatment groups, highlighting the protective effect of quercetin against Aβ_1-42_-induced oxidative stress. Data are presented as mean ± standard deviation (SD) from three independent experiments. Statistical significance is denoted as follows: ns (not significant) > 0.05, *p < 0.05, **p < 0.005, ***p < 0.0005.

Oxidative stress, a key driver in AD progression is known to both promote amyloid-β generation and intensify AD pathology. This study investigated the impact of quercetin on Aβ_1-42_ induced ROS production in SH-SY5Y cells using H_2_DCFDA staining and flow cytometry. Exposure of cells to 20 μM Aβ_1-42_ for 24 h resulted in elevated ROS levels. However, co-treatment with 100 μM quercetin significantly attenuated Aβ_1-42_-induced ROS production (Figures 7A–D). These findings indicate that quercetin effectively mitigated Aβ_1-42_ induced ROS generation. The reduction in H_2_DCFDA fluorescence intensity in the co-treated group reflects this decrease in cellular ROS, indicating that protective action of quercetin may be partly attributed to its ability to inhibit ROS generation.

### Effects of quercetin on MMP

**FIGURE 8:**
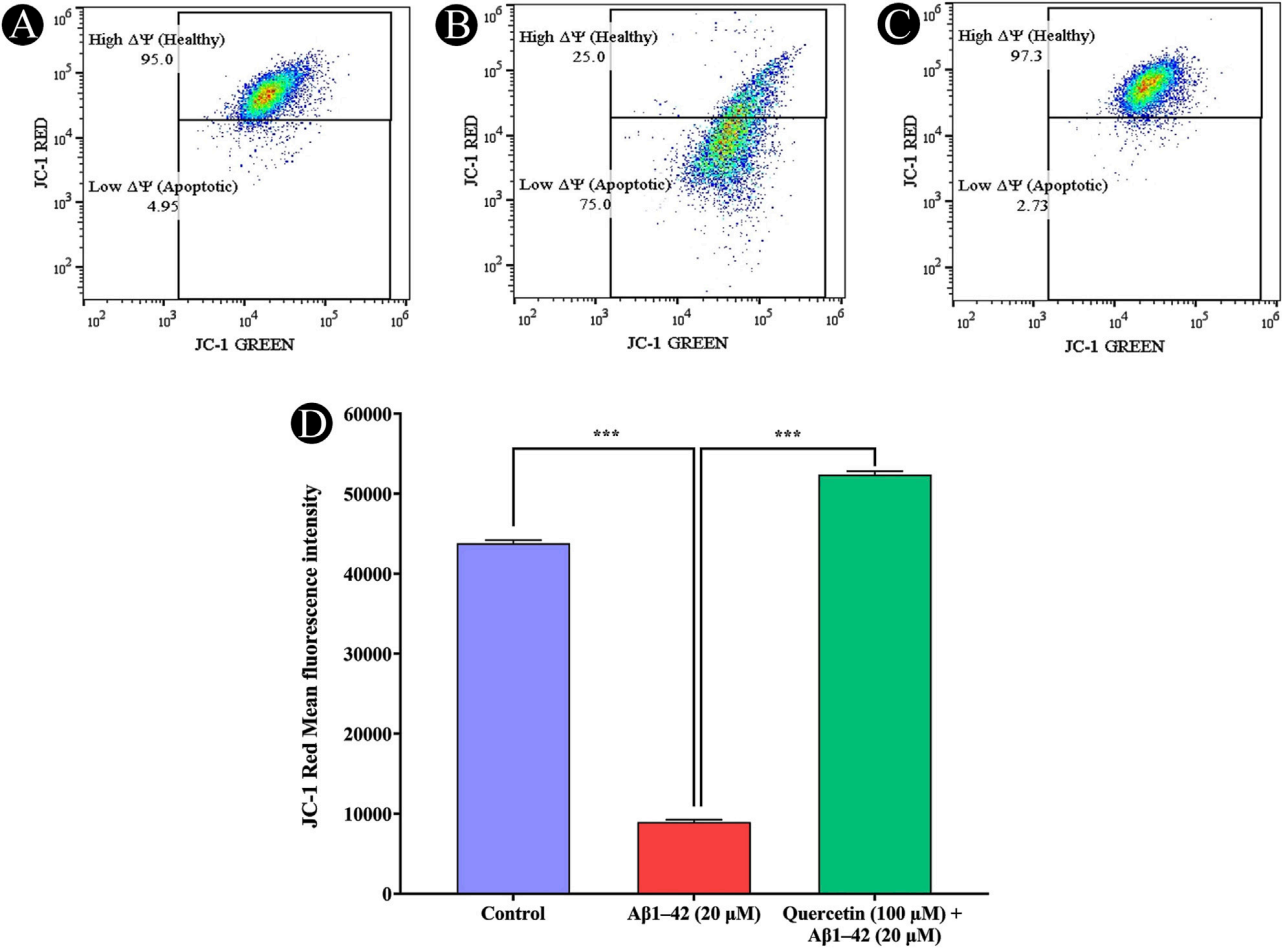
Analysis of mitochondrial membrane potential (Δψm) using JC-1 staining in SH-SY5Y cells. **(A)** Control cells exhibiting intact mitochondrial membrane potential, **(B)** Cells exposed to Aβ_1-42_ (20 µM), showing significant mitochondrial membrane depolarization, **(C)** Cells pre-treated with quercetin (100 µM) before Aβ_1-42_ (20 µM) exposure, demonstrating restoration of mitochondrial membrane integrity, **(D)** Quantitative analysis of JC-1 red fluorescence intensity, reflecting mitochondrial health across different treatment groups. Data are presented as mean ± standard deviation (SD) from three independent experiments. Statistical significance is denoted as follows: ns (not significant) > 0.05, *p < 0.05, **p < 0.005, ***p < 0.0005.

The mitochondrial membrane potential (Δψm), a key indicator of mitochondrial health, was evaluated in SH-SY5Y cells using JC-1 dye. Cells exposed to 20 μM Aβ_1-42_ exhibited a marked increase in mitochondrial depolarization, evidenced by decreased red fluorescence and increased green fluorescence, indicating mitochondrial dysfunction. Flow cytometry analysis confirmed a significant reduction in red fluorescence mean intensity and an elevated proportion of cells in the apoptotic region. However, pre-treatment with 100 μM quercetin effectively preserved mitochondrial membrane potential, as indicated by enhanced red fluorescence intensity and a reduced population of depolarized cells. These findings suggest that quercetin mitigates Aβ_1-42_ induced mitochondrial damage, thereby supporting mitochondrial integrity and enhancing cell viability (Figures 8A–D).

### Fluorescence microscopy assessment of JC-1-stained cells

**FIGURE 9:**
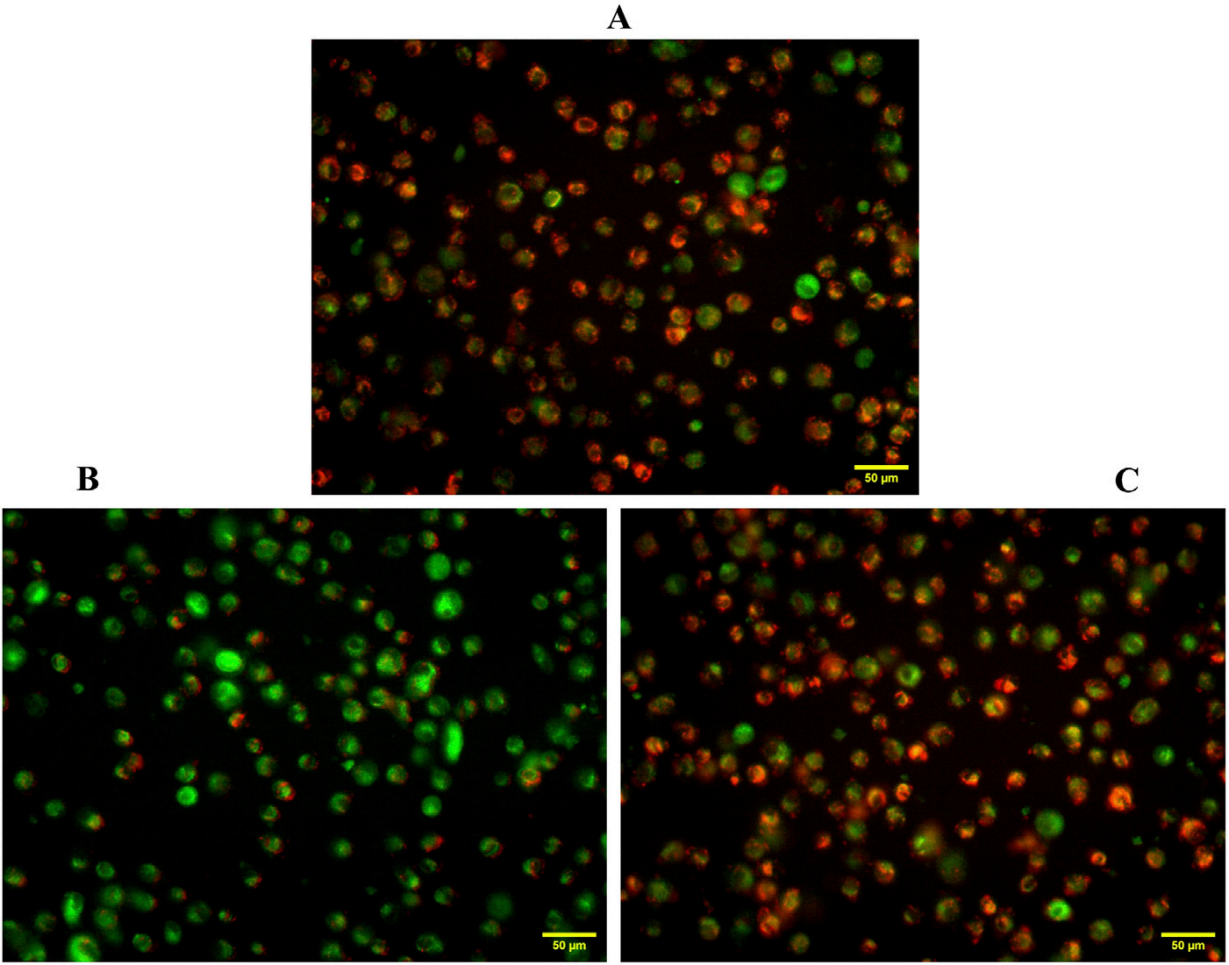
Fluorescence imaging of mitochondrial membrane potential in SH-SY5Y cells using JC-1 staining. **(A)** Untreated control cells exhibiting predominant red fluorescence, indicating healthy, polarized mitochondria, **(B)** Aβ_1–42_ treated cells displaying increased green fluorescence, indicative of mitochondrial depolarization and loss of Δψm, **(C)** Quercetin-treated cells showing restored red fluorescence, suggesting protective effects against Aβ_1–42_-induced mitochondrial dysfunction.

Mitochondrial membrane potential (Δψm) was further assessed using JC-1 staining in SH-SY5Y cells and visualized under a fluorescence microscope. In control (untreated) cells, strong red fluorescence was observed, indicating the presence of J-aggregates formed by JC-1 in polarized, healthy mitochondria, which is characteristic of live and metabolically active cells. In contrast, cells treated with Aβ_1-42_ alone exhibited a marked increase in green fluorescence and a significant reduction in red fluorescence, suggesting mitochondrial depolarization, a hallmark of early apoptosis and cell death. This shift from red to green fluorescence reflects a loss of Δψ and indicates compromised mitochondrial function in dying or dead cells. Notably, cells pre-treated with the quercetin prior to Aβ_1-42_ exposure showed a restoration of red fluorescence along with a reduction in green fluorescence, signifying the maintenance of Δψ, and a higher proportion of live and healthy cells. These observations further demonstrate that quercetin protects against Aβ_1-42_-induced mitochondrial dysfunction and promotes cell survival (Figures 9A–C).

## Discussion

The gradual loss of neuronal function and subsequent cell death frequently observed alongside Aβ peptide accumulation, characterizes neurodegenerative diseases, notably AD. The pathology of AD is predominantly influenced by the aggregation and misfolding of Aβ, leading to cellular toxicity and neuroinflammation (Cheignon et al., 2018; Jia et al., 2019; Piancone et al., 2021). Increasing evidence highlights the critical role of various environmental and molecular factors contributing to the progression of neurodegenerative changes, necessitating effective therapeutic interventions (Vyawhare et al., 2023; Khoury et al., 2024). Current study investigated the neuroprotective potential of quercetin, a natural flavonoid, towards Aβ-induced toxicity in human neuroblastoma SH-SY5Y cells. The obtained results demonstrate that quercetin exhibited significant neuroprotective activity via multiple mechanisms, including antioxidant activity, AChE inhibition, prevention of Aβ aggregation, mitochondrial membrane stabilization, and ROS modulation.

Aβ-induced neurotoxicity is primarily mediated by oxidative stress, mitochondrial dysfunction and cholinergic deficits, which contribute to synaptic failure and neuronal death. The accumulation of Aβ peptides in the brain is closely linked to increased oxidative stress, which elevates ROS and leads to significant cellular damage (Ba et al., 2022; Varesi et al., 2023). The results of the present study show that quercetin significantly enhances cell viability in Aβ-treated SH-SY5Y cells, suggesting its potential to counteract Aβ-induced cytotoxicity. Quercetin has been documented for its antioxidant properties, effectively neutralizing excessive ROS production, a major contributor to neuronal damage in AD (Ayvaz, 2019; Varesi et al., 2023). The antioxidant activity of quercetin was further supported by the H_2_DCFDA staining assay, which revealed a marked reduction in intracellular ROS levels upon quercetin treatment. This reduction in ROS mitigates oxidative stress and supports in the preservation of mitochondrial integrity, as mitochondrial dysfunction is a critical aspect of neurodegenerative diseases (Wang et al., 2020). By stabilizing mitochondrial function and preventing ROS accumulation, quercetin may help in the protection of neuronal health against the toxic effects of Aβ (Wang et al., 2020). Moreover, the combined properties of quercetin, including its potential to inhibit AChE, and its role in modulating mitochondrial processes, create a multifaceted approach to neuroprotection in the context of AD (Rahman et al., 2020). Additionally, ability of quercetin to maintain cellular homeostasis in the presence of Aβ-induced stress indicates its therapeutic prospects. It not only reduces oxidative damage, but also assists in mitigating cholinergic deficits, thereby further improving synaptic integrity and neuronal communication (Hakvoort et al., 2021). As oxidative stress plays a central role in these pathophysiological mechanisms, the ability of quercetin to act as both a direct scavenger of ROS and an enhancer of antioxidant pathways highlights its potential utility in AD management (Zhao et al., 2019; Mandal et al., 2023).

Cholinergic dysfunction is another crucial element in the pathology of AD, primarily due to excessive AChE activity. Our study determined that quercetin exhibits substantial AChE inhibitory activity, which could contribute to the restoration of cholinergic neurotransmission. Targeting AChE inhibition is a recognized therapeutic avenue for AD, as it enhances synaptic acetylcholine availability, and may improve cognitive function (Uddin et al., 2020; Jain et al., 2022). The capability of quercetin to modulate AChE activity suggests its potential as a promising candidate for AD intervention in agreement with the reported studies that flavonoids can act as multifaceted neuroprotective agents (Qi et al., 2020; Islam et al., 2021). Furthermore, the formation and aggregation of Aβ peptides into toxic oligomers and fibrils play a crucial role in AD pathogenesis. The results of Thioflavin T (ThT) assay demonstrated that quercetin effectively inhibits Aβ aggregation, indicating its ability to interfere with the fibrillization process. This anti-amyloidogenic property is significant, as preventing Aβ aggregation can reduce the formation of neurotoxic plaques and mitigate their detrimental effects on neuronal cells (Uddin et al., 2020; Jain et al., 2022; Li et al., 2022). The present findings are in agreement with previous work showing anti-amyloidogenic properties of flavonoids in AD models (Islam et al., 2021; Zhang et al., 2022).

Mitochondrial dysfunction is another hallmark of Aβ toxicity, leading to energy deficits and apoptotic cell death. The mitochondrial membrane potential (∆Ψm) assay in our study showed that quercetin treatment helps maintain mitochondrial integrity in Aβ-treated SH-SY5Y cells. The preservation of ∆Ψm suggests that quercetin prevents mitochondrial depolarization, a key indicator of mitochondrial dysfunction (Tsai et al., 2022; Fadzil et al., 2023; Oso et al., 2023). This protective effect on mitochondria may contribute to enhanced neuronal survival and resilience against Aβ toxicity, emphasizing its multifaceted role in neuroprotection (Oso et al., 2023; Sanad et al., 2023; Kerna et al., 2024).

Overall, the neuroprotective effects of quercetin observed in this study can be attributed to its multifunctional properties, including its ability to scavenge free radicals, inhibits AChE, prevent Aβ aggregation, and protect mitochondrial function. These findings are consistent with existing literature, which highlights the role of flavonoids in neuroprotection through their antioxidant, anti-inflammatory and anti-amyloidogenic mechanisms (Evans et al., 2022; Mandal et al., 2023; Sanad et al., 2023). Quercetin is well known for its notable neuroprotective properties, exerting its effects through various pathways, including the regulation of apoptosis, modulation of the oxidative stress response, and enhancement of neurotrophic factor activity. One of the primary mechanisms through which quercetin exerts its neuroprotective effects involves the regulation of apoptotic pathways, particularly through modulation of Bcl-2 family proteins. Studies indicate that quercetin decreases the expression of pro-apoptotic Bax while increasing the anti-apoptotic Bcl-2, thereby preventing neuronal death induced by oxidative stress and excitotoxicity (Yang et al., 2013). Specifically, the Western blot analyses conducted on hippocampal neuronal cell lines illustrate that quercetin enhances Bcl-2 levels while reducing cytochrome c release associated with mitochondrial permeability (Yang et al., 2013; Singh et al., 2024). This highlights the role of quercetin in protecting against neuronal loss due to apoptotic stimuli.

Additionally, quercetin is known to activate crucial neuroprotective transcription factors such as Nrf2, which plays a prominent role in the antioxidant response. Nrf2 activation regulates the expression of various genes involved in antioxidant defense and detoxification systems (Li et al., 2016). In a study involving human aortic endothelial cells, quercetin was demonstrated to enhance Nrf2 levels, suggesting that this transcription factor mediates its neuroprotective effects through the regulation of antioxidant enzymes and cellular detoxification pathways (Li et al., 2016). Furthermore, Nrf2 engagement is complemented by its interactions with other signaling molecules like p38 MAPK indicating a complex network of regulatory mechanisms (Li et al., 2016).

Quercetin also influences the expression of neurotrophic factors such as brain-derived neurotrophic factor (BDNF). Elevated levels of BDNF have been associated with improved neuronal survival, growth and synaptic plasticity, which are critical for restoration of neurodegenerative processes (Rahvar et al., 2018). This mechanism is particularly relevant in the context of diseases characterized by neuronal degeneration, as BDNF has been shown to enhance synaptic function and provide neuroprotection (Rahvar et al., 2018). The action of quercetin is also reported against neuroinflammation, an important feature of neurodegenerative diseases which is mediated by the inhibition of cyclooxygenase-2 (COX-2) and inducible nitric oxide synthase (iNOS), reducing pro-inflammatory cytokine release (Yang et al., 2018). Such effects link directly the influence of quercetin on the Wnt signaling pathway, specifically the canonical Wnt pathway, which has been implicated in neuroinflammatory responses (Yang et al., 2018). By modulating these pro-inflammatory pathways, quercetin contributes to a decrease in oxidative stress and neuroinflammation, further supporting neuronal survival.

Moreover, Heme Oxygenase-1 (HO-1) is a crucial cytoprotective enzyme that aids in defending against oxidative damage by catalyzing the degradation of heme into biliverdin, carbon monoxide, and iron, thus playing a significant role in cellular antioxidant defense mechanisms. The expression and activation of HO-1 are closely linked to the Nrf2 signaling pathway, which is a principal regulator of cellular responses to oxidative stress (Hu et al., 2021; Guo et al., 2022). Enhanced Nrf2 activation leads to the upregulation of antioxidant genes, including HO-1 and ultimately contributes to cell survival in response to oxidative damage (Robaczewska et al., 2016; Ji et al., 2021). In various pathological contexts, including neurodegenerative diseases and inflammation, the induction of HO-1 appears to be a protective response aimed at mitigating oxidative stress. For example, studies have shown that under oxidative conditions, HO-1 expression is significantly upregulated, which correlates with reduced levels ROS and subsequent cellular protection (Robaczewska et al., 2016; Ji et al., 2021; Mi et al., 2022). Conversely, decreased expression of HO-1 has been associated with degraded outcomes in oxidative stress-related diseases, suggesting its essential role in cytoprotection against oxidative injuries (Amin et al., 2014; Robaczewska et al., 2016).

The integration of Nrf2 and HO-1 pathways is particularly vital as they represent a feedback mechanism that reinforces cellular resilience during oxidative stress. When HO-1 is expressed, it not only helps to detoxify ROS but also modulates numerous downstream signaling pathways associated with inflammation and apoptosis (Amin et al., 2014; Robaczewska et al., 2016; Ji et al., 2021). Specifically, the Nrf2/HO-1 axis is found to be an essential component in various stress-responsive pathways, which include mitigating DNA damage and promoting cell survival (Miller et al., 2019; Khan et al., 2024). Addressing the status of HO-1 in human system is, therefore, crucial. In the present study, the HO-1 expression levels under conditions of induced oxidative stress via quercetin could elucidate its potential protective mechanisms within the mitochondrial context. Further exploration of upstream regulators like Nrf2, and their post-translational modifications that could alter HO-1 expression, will provide a comprehensive understanding of the interplay between these pathways in response to oxidative challenges. Although the findings are promising, it is crucial to recognize the limitations of this investigation. The *in-vitro* design fails to replicate the intricate complexities of AD pathology, as it occurs in living systems. Future studies should focus on validating these findings using animal models and clinical studies to establish the pharmacokinetics, bioavailability and long-term effects of quercetin in neurodegenerative conditions (Chen et al., 2023). Additionally, exploring the synergistic effects of quercetin with existing AD treatments may provide further insights into its therapeutic potential (Tsai et al., 2022; Kerna et al., 2024).

Moreover, quercetin currently faces several well-documented challenges that limit its clinical application, particularly in neurological disorders. Among the most prominent issues are its poor bioavailability, extremely low aqueous solubility (∼0.1 μg/mL at physiological pH), rapid degradation during gastrointestinal digestion and limited permeability across the blood–brain barrier (BBB) (Rich et al., 2017; Chen et al., 2018; Mukherjee et al., 2019). Its low solubility severely restricts gastrointestinal absorption, while its instability during digestion and storage further diminishes its therapeutic potential (Batiha et al., 2020; Jeayeng et al., 2025). Moreover, due to its hydrophilic nature and molecular structure, quercetin exhibits limited translocation across the BBB, hindering its effectiveness in central nervous system applications (Rich et al., 2017). To address these limitations, researchers have explored various nanoformulation strategies. Self-nanoemulsifying drug delivery systems (SNEDDS) have been shown to enhance solubility and absorption of quercetin by maintaining it in supersaturated states in the GI tract (Tran et al., 2014; Muhtadi et al., 2022). Additionally, lipid-based nanoparticles and encapsulation in biodegradable carriers have demonstrated promise in improving both stability and CNS delivery (Kumari et al., 2010; Hussain et al., 2021; Patel et al., 2024; Wang et al., 2024). These innovative approaches are critical for overcoming the inherent pharmacokinetic barriers and advancing quercetin’s viability as a therapeutic agent.

Overall, present study offers a comprehensive mechanistic investigation into the neuroprotective effects of quercetin against Aβ-induced toxicity in human SH-SY5Y neuroblastoma cells, setting it apart from previous studies that typically focus on isolated pathways. Unlike earlier studies, the present study simultaneously evaluates the impact of quercetin on oxidative stress, mitochondrial membrane potential, Aβ aggregation and AChE activity, four key pathological features associated with AD. By integrating these endpoints within a single experimental framework, our study addresses a critical gap in the literature regarding the multitargeted potential of natural compounds. Furthermore, the pharmacological limitations of quercetin highlighting the translational importance of the outcomes of this study in guiding future *in-vivo* studies and the development of advanced delivery systems. This integrated approach highlights the therapeutic relevance of quercetin and emphasizes the necessity of exploring plant-based multitarget agents for complex neurodegenerative conditions like AD.

## Conclusion

The present study provides compelling evidence for the neuroprotective role of quercetin against Aβ-induced toxicity in SH-SY5Y cells. By mitigating oxidative stress, inhibiting AChE activity, preventing Aβ aggregation and preserving mitochondrial function, quercetin demonstrates a multifaceted approach to neuroprotection. These findings support its potential as a promising natural therapeutic candidate for AD and other neurodegenerative disorders. However, further research is needed to explore its clinical applicability and efficacy in treating neurodegenerative diseases.

## Data Availability

The original contributions presented in the study are included in the article/supplementary material, further inquiries can be directed to the corresponding author.
